# An unusual case of Clostridium tetani bacteraemia likely arising from a skin and soft tissue infection

**DOI:** 10.1099/acmi.0.001177.v3

**Published:** 2026-08-20

**Authors:** Jun Yu Tay, Ibakkanavar Rajiva Guddappa, Alicia X. Y. Ang, Angie Natalie Pinto

**Affiliations:** 1 Department of General Medicine, Woodlands Hospital, Singapore, Singapore; 2 Department of Renal Medicine, Woodlands Hospital, Singapore, Singapore; 3 Department of Infectious Diseases, Woodlands Hospital, Singapore, Singapore

**Keywords:** bacteraemia, *Clostridium tetani*, skin and soft tissue infection

## Abstract

*Clostridium tetani*, the causative organism of tetanus, is unusual in developed countries, including Singapore, and to our knowledge, only three known cases of *C. tetani* bacteraemia have been reported worldwide. This case report describes a case of *C. tetani* bacteraemia in a Singaporean patient without clinical manifestations of tetanus, likely arising from a sacral sore, highlighting the need for early recognition and targeted antimicrobial treatment for the condition and its complications.

**Impact Statement**

This is the first case report in Singapore highlighting a case of *Clostridium tetani* bacteraemia, and only the fourth reported worldwide. This case serves as a reminder that even in highly vaccinated populations, *C. tetani* retains its capacity for invasive disease through alternative pathogenic mechanisms.

## Data Summary

In line with the Open Data policy of the journal, the following data have been published and made publicly available on Figshare: Identifier: https://doi.org/10.6084/m9.figshare.30655379.

## Introduction

*Clostridium tetani* is an anaerobic, spore-forming bacillus that is mainly transmitted through cuts, puncture wounds and animal bites that have been contaminated with dirt, faeces, soil and saliva [1]. It can also colonize the human gastrointestinal tract [1].

The pathogenesis of *C. tetani* has mainly been attributed to its toxin, which is taken up by the lower motor neurons of the peripheral nervous system and can ultimately reach the central nervous system. The toxin then interferes with inhibitory neurotransmitters in the nervous system, such as gamma-aminobutyric acid and glycine, resulting in the clinical manifestations of tetanus, such as trismus, risus sardonicus, dysphagia, neck stiffness, abdominal rigidity and opistotonus [2].

In Singapore, vaccinations against *C. tetani* have been incorporated into the national immunization schedule since 1959 [3]. At present, all Singaporeans receive three doses of the Diphtheria, Tetanus and acellular Pertussis vaccine, one booster dose and one dose of Tetanus, reduced diphtheria and acellular pertussis content vaccine. Pregnant women are recommended to receive the Tdap vaccine between 16 and 32 weeks of pregnancy. As part of post-exposure prophylaxis, the tetanus toxoid vaccine is also routinely administered for exposed wounds [4].

## Case presentation

A 63-year-old Singaporean gentleman with a 20-year history of alcohol dependence was brought to the Emergency Department having been found by his daughter on the floor of his bedroom in a stuporous state. It was unclear how long he had been on the ground as the patient was unable to provide any meaningful history other than complaining of bilateral hip pain. Corroborative history obtained suggests that prior to admission, he had been living alone and was last seen leaving his residence by his neighbours about 1 week prior. It was also noted that he was frequently observed to be intoxicated by his neighbours.

It was noted by paramedics that he had an altered mental status and hypotension of 75/54 mmHg. On presentation to the Emergency Department, he was tachycardic with a heart rate of 123 beats per minute and febrile with a temperature of 38 °C.

On initial examination, the patient was clinically dry, dehydrated and disoriented to place with a Glasgow Coma Scale of 11 (E4V2M5). Notably, a stage 4 sacral sore measuring 10 cm in length was identified. There was also tenderness over the bilateral hips, although the ranges of motion over his bilateral knees and ankles were intact. Tone in the upper limbs was normal, while the lower limb neurological examination was limited due to pain.

Laboratory tests on admission (consolidated in Table 1) yielded an elevated white blood cell count of 19.1×10^9^/L and raised inflammatory markers, including a C-reactive protein level of 308.3 mg/L and a procalcitonin level of 1.38 μg/L. Creatine kinase was elevated at 2,000 U/L but subsequently normalized on day 9 of admission to 114 U/L. Evidence of acute kidney injury was also present on admission, with elevated serum urea at 27.8 mmol/L and serum creatinine at 164 μmol/L, both of which normalized with adequate fluid therapy by day 5 of admission and remained within normal limits for the remainder of the patient’s hospital stay. Aspartate transaminase was raised at 119 U/L, while other liver enzymes were in the normal range. Serum lactate, electrolyte levels and his coagulation profile were all within the normal range.

**Table 1.** Selected laboratory test results of the patient with normal reference ranges, in keeping with a clinical picture of sepsis, acute kidney injury and rhabdomyolysis from possible long lie

Selected laboratory test results on admission		Reference ranges
White blood cell count	19.1×10^9^/L	4.0–9.6×10^9^/L
C-reactive protein (CRP)	308.3 mg/L	0.0–5.0 mg/L
Procalcitonin	1.38 ug/L	0.00–0.06 μg/L
Creatinine kinase	2000 U/L	50–350 U/L
Urea	27.8 mmol/L	2.5–7.5 mmol/L
Creatinine	164 umol/L	60–105 μmol/L
Aspartate transaminase (AST)	119 U/L	15–40 U/L
Lactate	1.5 mmol/L	0.5–2.2 mmol/L
Sodium	142 mmol/L	135–145 mmol/L
Potassium	4.3 mmol/L	3.5–5.0 mmol/L
Calcium (corrected)	2.33 mmol/L	2.15–2.50 mmol/L
Phosphate	0.8 mmol/L	0.8–1.4 mmol/L
International normalized ratio (INR)	1.0	–

A computed tomography (CT) scan of the brain yielded no evidence of intracranial haemorrhage or large territorial infarction, and no fractures were picked up on skeletal survey. Blood and urine cultures were taken, and the patient was started empirically on intravenous (IV) amoxicillin–clavulanic acid 1.2 g every 8 h. He was subsequently admitted to the general ward.

Blood cultures returned positive 24 h after collection, with growth of *Enterococcus* species, and the antibiotic regimen was switched to IV vancomycin with a loading dose of 25 mg kg^−1^ followed by 1 g every 24 h. Infectious diseases were consulted, and the likely source of bacteraemia was localized to the sacral injury. Preliminary results of blood cultures after 48 h yielded *Enterococcus gallinarum* and *Enterococcus raffinosus*. Susceptibility testing was performed using Vitek^®^ antimicrobial susceptibility testing according to Clinical and Laboratory Standards Institute standards. *E. gallinarum* is inherently resistant to vancomycin but was sensitive to ampicillin. *E. raffinosus* was resistant to ampicillin but sensitive to vancomycin. Thus, IV ampicillin 2 g every 8 h was commenced for the former, and IV vancomycin, with regular trough levels measured, was continued to treat the latter. Tissue cultures from the buttocks yielded a mixed bacterial growth, including *Escherichia coli*. There was no bacterial growth arising from the urine culture.

**Fig. 1.**
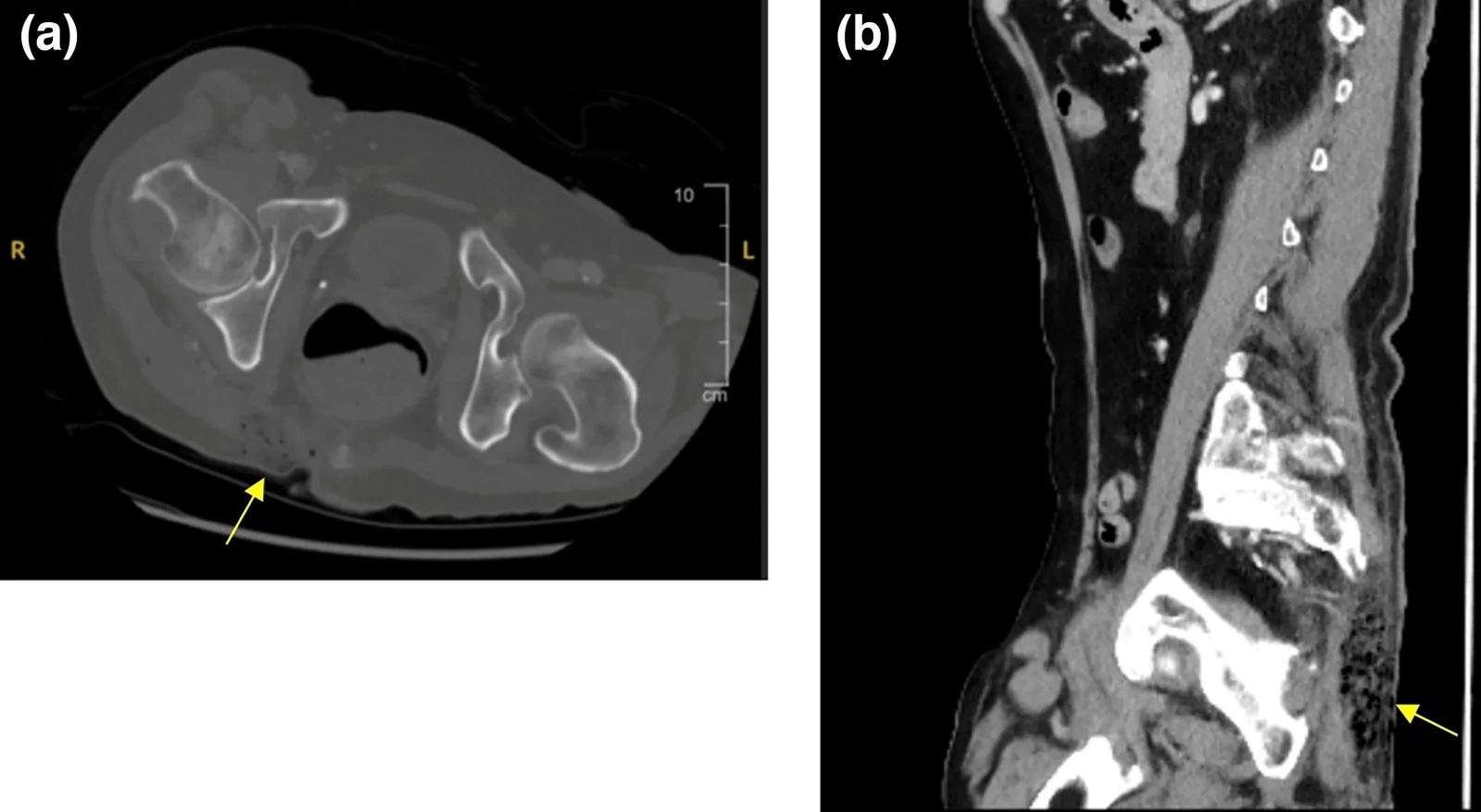
(**a**) Axial and (**b**) Sagittal cuts on the patient’s CT scan showing a large right sacral wound with surrounding inflammatory changes as well as gas locules and tracks in the soft tissue.

**Fig. 2.**
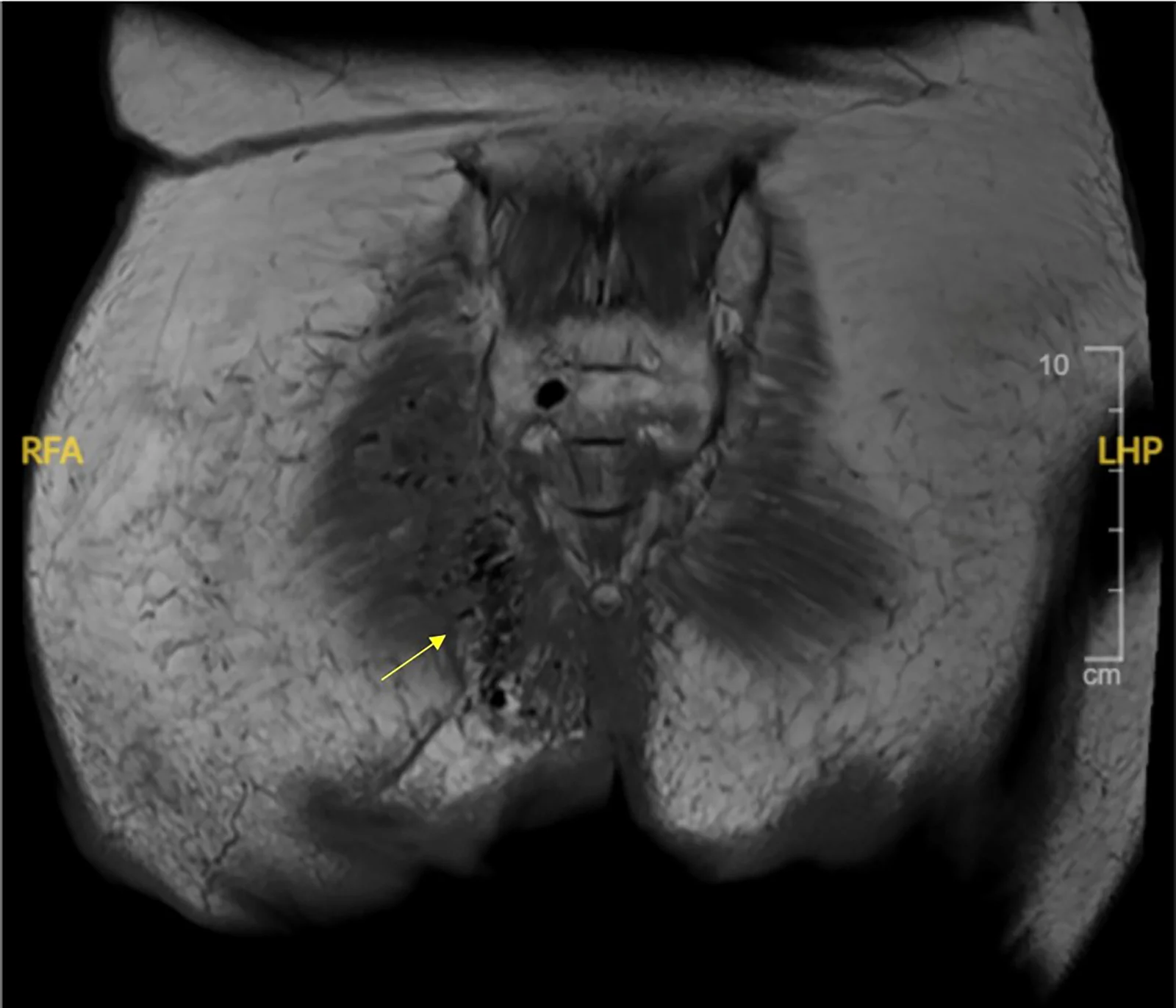
T1-weighted MRI of the patient’s pelvic region demonstrating a large and deep right sacral sore involving the right gluteus maximus muscles and causing lower sacral early osteomyelitis on the right. This was also associated with gas locules (arrow) and diffuse oedema in surrounding muscle groups consistent with myositis.

Repeat blood cultures collected on days 3 and 5 of admission yielded no bacterial growth, indicating the clearance of bacteraemia. However, a CT scan of the thorax, abdomen and pelvis (CTTAP) revealed a large right sacral wound with surrounding inflammatory changes, including underlying gas locules at the right posterior sacral bone and gas tracks along some of the fibres of the right gluteus maximus muscle (Fig. 1). Vascular surgery and orthopaedic surgery were consulted, and the patient was deemed not amenable for surgical debridement in view of the inability to commit to the likelihood of requiring multiple surgeries, high nursing requirements, and a prolonged period of offloading and eventual need for flap coverage. Subsequent magnetic resonance imaging (MRI) of the pelvis demonstrated a sacral wound with non-enhancing soft tissue and gas extending inferiorly and laterally along the gluteus maximus muscle (Fig. 2). Antibiotic therapy was hence continued to treat the patient for his stage 4 sacral ulcer with right gluteal myositis and sacral osteomyelitis. Specialized nursing for wound care was initiated, and conservative sharp wound debridement was performed for the patient.

Eight days after the initial blood cultures were taken, there was growth of *C. tetani* on CDC anaerobic blood agar plates. Identification was confirmed by MALDI-TOF MS with a score of 2.24, and Gram stain confirmed distinctive morphology with ‘drumstick’ or ‘tennis racket’-shaped Gram-positive rods; thus, oral metronidazole was added. Notably, the patient displayed no clinical signs of muscle or jaw spasms, neck stiffness or dysphagia. Muscle tone was normal. He had received an intramuscular TETAVAX tetanus toxoid vaccine a little more than a year ago, when he had presented with a fall with a left brow laceration. His past medical history was also negative for any previous diagnosis of tetanus. Therefore, tetanus immunoglobulin was not indicated as the patient was not deemed to have clinical features consistent with tetanus. As local public health authorities only require clinical notification of tetanus and laboratory notification was not required, no further referral or testing of the isolate was performed.

**Fig. 3.**
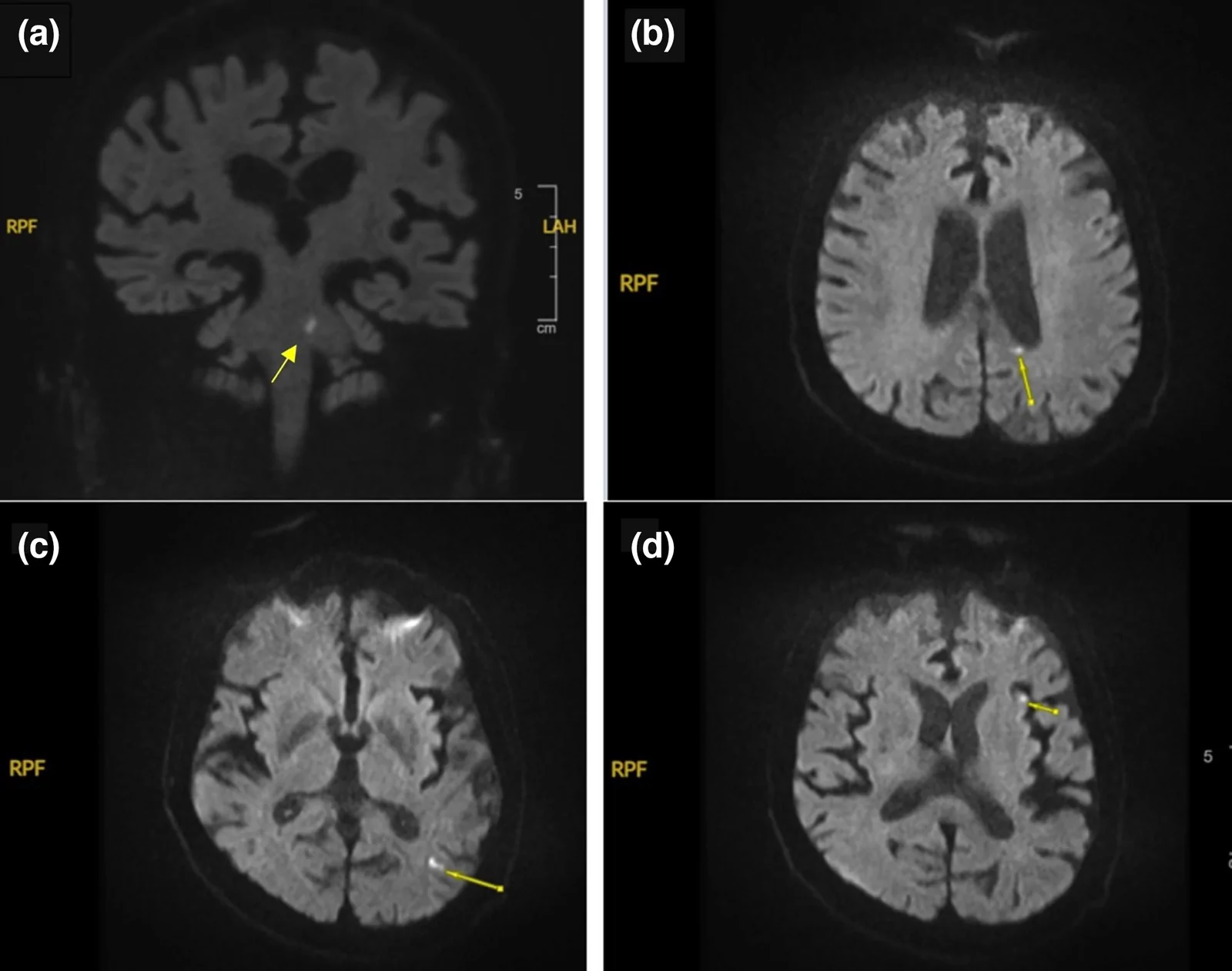
Diffusion-weighted imaging MRI of the patient’s brain showing small foci of acute infarcts in the (**a**) left hemi-pontine region, (**b**) left periventricular region, (**c**) left parietal lobe and (**d**) left insula. These possibly point to the presence of septic emboli, a minor criterion in the Duke criteria for infective endocarditis.

In the following weeks, IV gentamicin 3 mg kg^−1^ every 24 h was added for 2 weeks to empirically treat for infective endocarditis given that two consecutive transthoracic echocardiogram (TTE) scans were suboptimal and unable to exclude this condition because of the patient’s non-cooperation and that three minor criteria in the Duke criteria had been fulfilled – fever, microbiological evidence and vascular phenomena in the form of multifocal infarcts in the brain with no large vessel occlusion or flow limiting stenosis (Fig. 3), possibly suggestive of septic emboli. It was noted that the patient had no significant history of cardiovascular conditions such as ischaemic heart disease or atrial fibrillation. The patient had also refused further forms of invasive investigations such as a transoesophageal echocardiogram. Nevertheless, serial blood cultures obtained on days 14 and 21 of admission continued to show no bacterial growth.

IV ampicillin was stopped after 6 weeks, while the patient continued to receive oral metronidazole and IV vancomycin and was discharged from our acute tertiary hospital to a community hospital on day 45 of admission for rehabilitation and continuation of antibiotics, planned for a total duration of 8 weeks. A consolidated summary of events can be found in Table 2.

**Table 2.** A summary of significant events over the course of the patient’s admission classified according to microbiology findings, antibiogram profile and relevant imaging results

Microbiology (day of collection)
Blood cultures Day 1 – *E. gallinarum, E. raffinosus, Clostridium tetani* Day 3 – no bacterial growth Day 5 – no bacterial growth Day 14 – no bacterial growth Day 21 – no bacterial growth Tissues cultures Day 1 – mixed bacterial growth including *E. coli*; no fungal growth Urine culture Day 1 – no bacterial growth
**Antibiogram**
Day 1 – IV Amoxicillin–clavulanic acid (1 day) Day 2 to day 44 – IV ampicillin (6 weeks) Started after blood cultures grew *E. gallinarum*, continued for treatment of possible infective endocarditis Day 2 onwards – IV vancomycin (planned for a total of 8 weeks duration) Started after blood cultures grew *E. raffinosus*, continued for treatment of possible infective endocarditis Day 9 onwards – PO metronidazole (planned for a total of 8 weeks duration) Started after blood cultures grew *C. tetani* Day 20 to day 34 – IV gentamicin (2 weeks) Started for treatment of possible infective endocarditis
**Imaging**
Day 1 – CT brain: no intracranial haemorrhage, no large territorial infarction Day 3 – CTTAP: large right sacral wound with surrounding inflammatory changes Day 3 – TTE: technically difficult study. Patient unable to be repositioned Day 4 – MRI brain stroke protocol: small foci of acute infarcts noted, possibly suggestive of infective endocarditis. No large vessel occlusion or flow-limiting stenosis Day 16 – MRI pelvis: large and deep right sacral sore, right gluteus maximus, early osteomyelitis and myositis Day 16 – Repeat TTE: technically difficult study. The patient was uncooperative and aggressive throughout

## Discussion

Worldwide, bacteraemia from *C. tetani* infection is exceedingly rare. A literature review of three databases – PubMed, MEDLINE Ovid and Embase – has found only three reported cases of *C. tetani* bacteraemia, two of which arose from likely intra-abdominal sources and the remaining one from a likely skin source [5–7]. The patients described by Hallit *et al*. and Kazadi *et al*. were up to date with their tetanus immunizations. Antibiotics with anaerobic coverage, such as metronidazole and vancomycin, were utilized, and clinical improvement followed in all three cases reported. The polymicrobial nature of infection in our case also necessitated a more complex antibiotic regimen.

All cases had possible evidence of immunocompromise or immunosuppression. The case by Kazadi *et al*. had mediastinal lesions concerning for neoplastic disease, although further work-up was declined, whereas the patient described by Lai *et al*. had hepatocellular carcinoma and had undergone trans-arterial embolization prior to developing bacteraemia. The case by Hallit *et al*. had received steroid therapy for a chronic obstructive pulmonary disease (COPD) exacerbation. Given our patient’s unusual bloodstream infection and history of chronic alcoholism predisposing him to functional immunosuppression, investigations were conducted to identify any additional underlying causes of immunocompromise. Screening for hepatitis viruses was unremarkable, the human immunodeficiency virus screen was non-reactive, and his haemoglobin A1c was 4.7%. There was no evidence of malignancy noted on CTTAP. A comparative analysis summary of the case reports is presented in Table 3.

**Table 3.** A summary of cases of *C. tetani* bacteraemia reported in the existing literature

Case	Case at hand	Kazadi *et al*. (2022)	Lai *et al*. (2017)	Hallit *et al*. (2012)
**Presentation**	63-year-old Singaporean man presenting with an unwitnessed fall	86-year-old man from USA presenting with an unwitnessed fall	73-year-old Taiwanese man with fever, chills and vomiting	87-year-old African American woman admitted for generalized weakness and diarrhoea
**Source of infection**	Stage 4 sacral ulcer with right gluteal myositis and sacral osteomyelitis	Left lower extremity cellulitis with ulceration and sacral decubitus ulcer with necrotic eschar	Trans-arterial embolization treatment for hepatocellular carcinoma 4 days prior	Non-reducible ventral hernia and colonoscopy-proven colitis
**Evidence of immuno-compromise/immuno-suppression**	20-year history of alcohol dependence	Mediastinal lesions concerning for neoplastic disease, further evaluation declined	Hepatitis-related liver cirrhosis and hepatocellular carcinoma	Recent admission for COPD exacerbation, treated with steroids and nebulizers
**Immunization status**	TETAVAX vaccine 1 year ago	TDaP vaccine received 7 years prior to presentation	Not discussed	Up to date
**Management and outcome**	IV ampicillin, IV vancomycin, PO metronidazole, IV gentamicin for infective endocarditis Discharged to a community hospital for rehabilitation after 45 days	4 weeks of antibiotics (IV piperacillin–tazobactam and IV vancomycin, subsequently PO ampicillin) Discharged to rehabilitation unit after 5 weeks of hospitalization	Sensitivity-guided metronidazole Followed up at the outpatient clinic	Booster dose of tetanus and diphtheria vaccine (Td) and oral metronidazole Surgery was declined

Similar to this case, none of the previously reported cases displayed clinical signs of tetanus. This differs from the typical cases of tetanus classically described in areas of the world where inadequate vaccination coverage remains the primary risk factor [1]. The reason for the lack of clinical manifestation of tetanus has been hypothesized to either be due to sufficient prior immunization resulting in successful neutralization of tetanus toxins or that the infecting bacterial strain did not produce toxins [5, 7].

This case highlights a critical diagnostic paradox: while tetanus vaccination programmes have virtually eliminated clinical tetanus in Singapore, the manifestation of bacteraemia likely arising from the skin and soft tissue portal of entry remains a rare manifestation. Given this organism’s notorious virulence, any positive blood culture warrants urgent assessment for occult infection sources and consideration of antimicrobial therapy, regardless of vaccination status.

## Conclusion

This case serves as a reminder that even in highly vaccinated populations, *C. tetani* retains its capacity for invasive disease through alternative pathogenic mechanisms. Prompt source control and, where indicated, immunoglobulin administration should be implemented in a timely fashion.

## Abbreviations

COPD: chronic obstructive pulmonary disease
CT: computed tomography
CTTAP: CT scan of the thorax, abdomen and pelvis
IV: intravenous
MRI: magnetic resonance imaging
TTE: transthoracic echocardiogram

## References

[1] George EK, De Jesus O, Tobin EH, Vivekanandan R (2025). Tetanus (*Clostridium tetani* infection). https://www.ncbi.nlm.nih.gov/books/NBK482484/.

[2] Hassel B (2013). Tetanus: pathophysiology, treatment, and the possibility of using botulinum toxin against tetanus-induced rigidity and spasms. Toxins (Basel).

[3] Goh KT (1985). The national childhood immunisation programmes in Singapore. Singapore Med J.

[4] Handbook on adult vaccination in Singapore. https://www.cfps.org.sg/assets/Circulars/2023/SG-SID-001-Handbook-v15-Highres-bleed.pdf.

[5] Hallit RR, Afridi M, Sison R, Salem E, Boghossian J (2013). *Clostridium tetani* bacteraemia. J Med Microbiol.

[6] Lai CC, Chen CC, Hsu HJ, Chuang YC, Tang HJ (2018). *Clostridium tetani* bacteremia in a patient with cirrhosis following transarterial chemoembolization treatment for hepatocellular carcinoma. J Microbiol Immunol Infect.

[7] Kazadi D, Zychowski D, Skipper C, Teravskis P, Hansen GT (2022). *Clostridium tetani* bacteremia from a suspected cutaneous source. Cureus.

